# Synonymous single nucleotide polymorphism in arsenic (+3) methyltransferase of the Western mosquitofish (*Gambusia affinis*) and its gene expression among field populations

**DOI:** 10.1007/s10646-021-02376-8

**Published:** 2021-04-03

**Authors:** Daesik Park, Catherine R. Propper, Guangning Wang, Matthew C. Salanga

**Affiliations:** 1 Division of Science Education, Kangwon National University, Chuncheon, Kangwon 24341 South Korea; 2 Department of Biological Sciences, Northern Arizona University, Flagstaff, AZ 86011 USA

**Keywords:** Arsenic, *as3mt*, *Cyt19*, mosquitofish, *Gambusia affinis*, Arizona

## Abstract

Naturally occurring arsenic is toxic at extremely low concentrations, yet some species persist even in high arsenic environments. We wanted to test if these species show evidence of evolution associated with arsenic exposure. To do this, we compared allelic variation across 872 coding nucleotides of arsenic (+3) methyltransferase (*as3mt*) and whole fish *as3mt* gene expression from three field populations of *Gambusia affinis*, from water sources containing low (1.9 ppb), medium-low (3.3 ppb), and high (15.7 ppb) levels of arsenic. The high arsenic site exceeds the US EPA’s Maximum Contamination Level for drinking water. Medium-low and high populations exhibited homozygosity, and no sequence variation across all animals sampled. Eleven of 24 fish examined (45.8%) in the low arsenic population harbored synonymous single nucleotide polymorphisms (SNPs) in exons 4 and/or 10. SNP presence in the low arsenic population was not associated with differences in *as3mt* transcript levels compared to fish from the medium-low site, where SNPs were noted; however, *as3mt* expression in fish from the high arsenic concentration site was significantly lower than the other two sites. Low sequence variation in fish populations from sites with medium-low and high arsenic concentrations suggests greater selective pressure on this allele, while higher variation in the low population suggests a relaxed selection. Our results suggest gene regulation associated with arsenic detoxification may play a more crucial role in influencing responses to arsenic than polymorphic gene sequence. Understanding microevolutionary processes to various contaminants require the evaluation of multiple populations across a wide range of pollution exposures.

## Introduction

Rapid evolutionary responses to contaminants occur through both changes in gene sequence and gene expression. Adaptation to various contaminants has recently been reported through comparing mutations and expression of specific genes associated with detoxification of pollutants across populations (Schlebusch et al. 2015; Reid et al. 2016; Gouin et al. 2019). Species introductions into contaminated areas provides an opportunity to evaluate early responses and/or adaptive processes to contaminants in the wild. Acquisition of new variations during the adaptation process at introduced sites often occur (Oziolor et al. 2019).

As well-known carcinogens, arsenics including inorganic arsenic (iAs), arsenate (iAs^V^), and arsenite (iAs^III^) are widely found in drinking water, food, and other environmental sources. These compounds are involved in various negative impacts on health and welfare in humans and wildlife (Hughes et al. 2011; Carlin et al. 2016; Minatel et al. 2018). Vulnerable animal populations living in arsenic contaminated water face selective pressure to maintain alleles which encode arsenic detoxifying enzymes, such as As3mt, which metabolizes ingested arsenic into lower-toxicity compounds (Zhang et al. 2012).

**Fig. 1:**
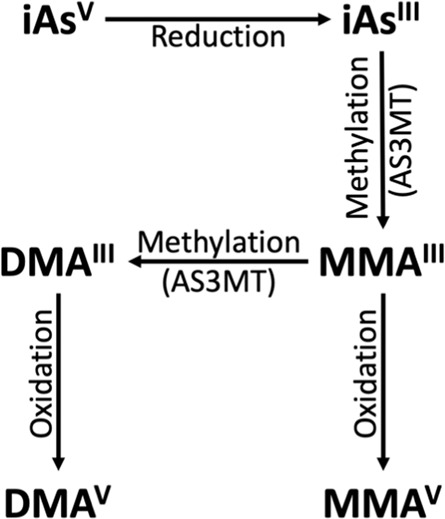
Diagrammatic representation of arsenic metabolic pathways. After reduction of arsenate (iAs^V^) into arsenite (iAs^III^), it is metabolized to monomethylarsonic acid (MMA^III^ or MMA^V^) and subsequently to dimethylarsinic acid (DMA^III^ or DMA^V^), a less toxic chemical, via methylation by arsenite (+3) methyltransferase (*as3mt*) and oxidation. The diagram is based on Khairul et al. (2017) and Minatel et al. (2018)

Arsenic (+3) methyltransferase (AS3MT; alias CYT19) is a key enzyme involved in the metabolic process of detoxifying iAs^V^ and iAs^III^ through methylation of the arsenicals to less toxic arsenic forms of monomethylarsonic acids (MMA^III^, MMA^V^) and dimethylarsinic acids (DMA^III^, DMA^V^) (Khairul et al. 2017; Minatel et al. 2018) (Fig. 1). Orthologues for *AS3MT* are present across vertebrate clades, and its function in mitigating arsenic toxicity has been observed in multiple laboratory test species, including zebrafish (Hamdi et al. 2012; Bambino et al. 2018), *Xenopus* (Koch et al. 2015), and rodents (Waters et al. 2004; Coryell et al. 2018; Stýblo et al. 2019). Additionally, a bioinformatic query revealed uncharacterized orthologues in many more species, including birds and reptiles (Hunt et al. 2018) (Fig. S1).

In humans, decreased activity of AS3MT is linked to a SNP (T > C), resulting in a Met287Thr amino acid change (Drobna et al. 2009). Several other studies show allelic variation within human populations (Schlebusch et al. 2015; Apata and Pfeifer 2020), with polymorphisms detected in the *AS3MT* 5ʹ-UTR that affect the conversion of inorganic arsenic to its organic forms (Wood et al. 2006; Valenzuela et al. 2009; Antonelli et al. 2014). Despite the widespread distribution of arsenic in nature, unlike studies in human populations, there are relatively few studies on whether mutations in *AS3MT* impact wildlife (Boyle et al. 2008; Ye et al. 2014; Hallauer et al. 2016). Such investigations in populations that have short generation times can lead to a better understanding of how *AS3MT* SNP distribution can impact AS3MT function as an evolutionary response to xenobiotic exposure.

Western mosquitofish (*Gambusia affinis*) have been widely used as a model animal to study the effects of various environmental pollutants (Turner 1942; Bortone and Davis 1994; Park et al. 2004). Western mosquitofish are native in the South-Central US and into Mexico, and are believed to have been introduced into Arizona in the early 1920s. They now inhabit 22 different Hydrologic Unit Code sites in the state (Dees 1961; Miller and Lowe 1967), though their population and genetic origins are undetermined. Surface waters in Arizona demonstrate considerable variation in arsenic contamination levels, with waterways below the current United States Environmental Protection Agency’s drinking water limit of 10 ppb (parts per billion), to stream and rivers containing arsenic concentrations well above 100 ppb (Jones et al. 2020). Considering that various negative effects of arsenic have been reported in fish (Boyle et al. 2008; Hallauer et al. 2016), and that nuclear genes have shown rapid evolution within 80–90 years following the introduction in other fish populations (Dlugosch and Parker 2008), it is possible that adaptive selection to metabolize or detoxify arsenic effectively may have occurred in *G. affinis*.

In this study, we tested the hypotheses that the identity of *as3mt* allele variants and their expression correlate with arsenic levels in that fish’s water resource. Like in human studies, we predict, that SNP containing *as3mt* alleles identified in *G. affinis* exposed to high arsenic will exhibit differences in frequencies related to their environmental arsenic exposure probability, and that expression of *as3mt* will track with arsenic levels in the water resources.

## Materials and methods

### Collecting sites

**Fig. 2:**
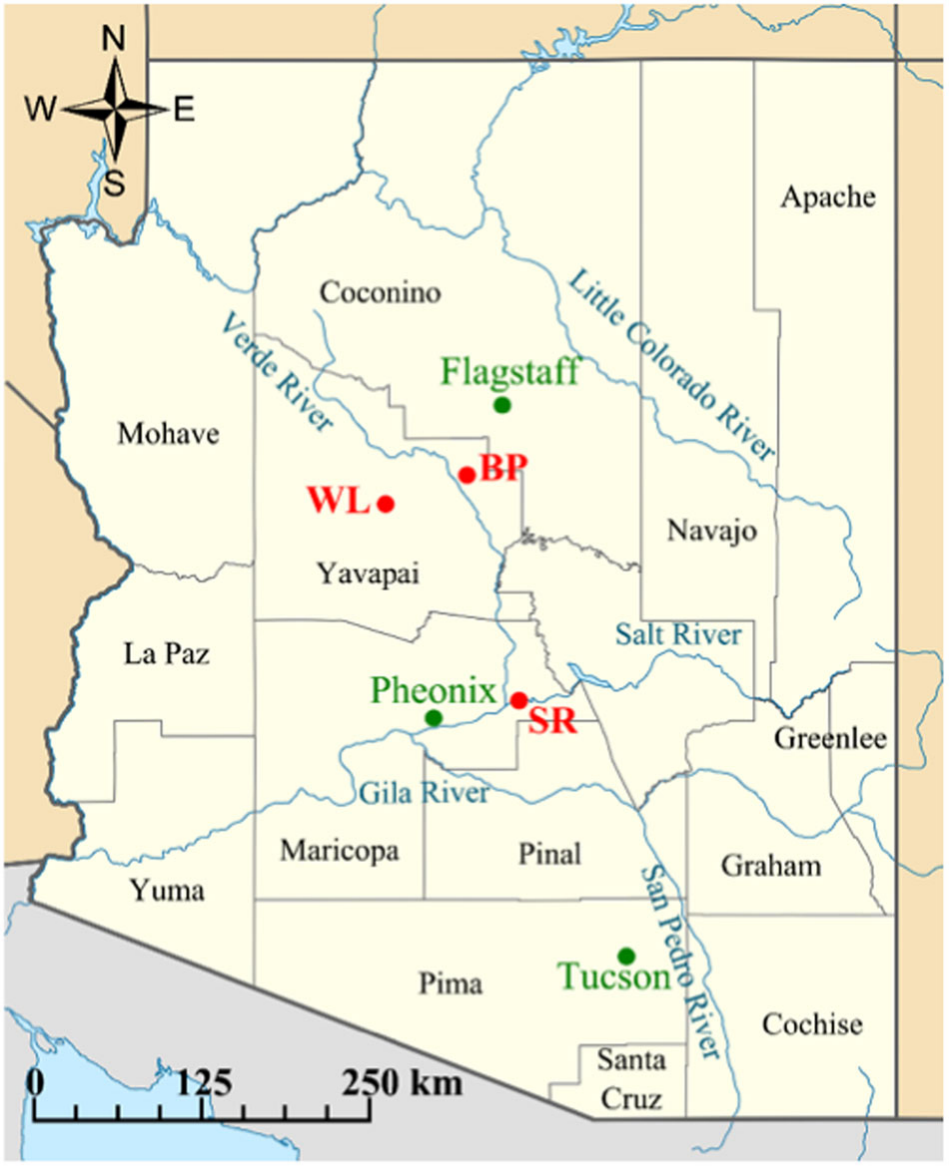
Map of Arizona and collection sites for Western mosquitofish. BP Bubbling Pond fish-hatchery, WL Willow Lake, SR Salt River

For the study, we collected Western mosquitofish (Arizona Game and Fish Department; LIC#SP649802) at the outflow ditch of Bubbling Pond fish-hatchery (BP, 17 males, 34.45 N, −111.53E), Willow Lake (WL, 24 males, 34.36 N, −112.26E), and the Salt River, just below Saguaro Lake (SR, 17 males, 33.33 N, −111.32E, Fig. 2) between March 30 and August 23, 2019 using dip nets. The fishes were euthanized in 0.04% buffered tricaine methanesulfonate (MS222, pH 7.0, CAS# 886-86-2), individually frozen on dry ice in the field, brought to the lab, and stored at −80 °C until use. To avoid potential interference caused by females’ reproductive status, we chose only males for further quantitative PCR. All protocols were approved under Northern Arizona University’s Institutional Animal Care and Use Committee (IACUC Protocol #19-013).

### Inductively coupled plasma mass spectrometry (adapted from Credo et al. 2019)

For this study, arsenic levels were quantified at only one time point for each site. We collected two 500 ml water samples from each of the three sites on December 4, 2019 in clean wide-mouth packers (#05-719-149B, Fisher Scientific). 100 ml of each water sample were filtered into two clean 50 ml Falcon tubes using a 0.45 μm Whatman uniflo syringe filter (#09-928-061, Fisher Scientific) and a Norm-ject 30 ml luer-lock pp/pe syringe (#14-817-41, Fisher Scientific). The filtered water samples were kept ice cold while transporting to the laboratory, where they were stored at 4 °C until analysis. The water samples were acidified with concentrated trace metal nitric acid to a pH of approximately 2.0. Acidified samples were diluted 1:10 with 2% trace metal HNO_3_ and spiked with an internal standard (Iridium and Rhodium) at 1.0 ppb. Prepared samples (two for each site) were run in triplicate on a Thermo X-Series II Inductively Coupled Plasma Mass Spectrometer (ThermoFisher). In parallel, calibration standards (spiked with the internal standard) and a certified reference material (NIST 1640a – Trace Metals in Natural Waters) were also run. Dissolved arsenic concentrations at each site were determined by averaging arsenic levels from two samples.

### RNA isolation and cDNA synthesis

To measure genotype specific *as3mt* gene expression, we isolated RNA from genotype verified individual fish for RNA isolation and gene expression analysis. Whole individuals were homogenized in 0.5–2 ml of TRIzol (#15596026, ThermoFisher) using a PowerGen 125 homogenizer (#NC0530997, Fisher Scientific) in microcentrifuge tubes. DNA-free total RNA was extracted from each sample using Direct-Zol RNA MiniPrep kit (#R2050-51, Zymo Research) following the company’s protocols. Isolated RNA concentration was quantified using a NanoDrop Lite (Thermo Scientific), inspected by gel electrophoresis for ribosomal RNA integrity, and cDNA was synthesized from 1 µg of DNA-free total RNA using BioRad iScript cDNA synthesis kit (#1708890, BioRad). Briefly, total reaction volume of 20 μL containing 1 μg of the extracted RNA was used, and the final mixture was incubated at 5 min at 25 °C, 20 min at 46 °C, 1 min at 95 °C, and holding at 4 °C in a thermocycler (SimpliAmp, Applied Biosystems). Synthesized cDNA samples were stored at −20 °C.

### PCR and allele sequencing

For PCR amplification of *as3mt*, we designed forward and reverse primers (Table 1) based on the *G. affinis as3mt* reference sequence obtained from Ensembl (ENSGAFG00000012739; Hunt et al. 2018), which consists of 1119 base pairs over 11 exons. One-twentieth of the total cDNA (1 ul) was used as template and amplified by Q5’ High Fidelity DNA polymerase (#M0491, New England BioLabs) in a 40 μl reaction volume according to manufacturer’s instructions. Briefly, the mixture was incubated at 10 s at 98 °C, 30 s at 58 °C, 45 s at 72 °C for 35 cycles in a PCR thermocycler (SimpliAmp, Applied Biosystems). PCR amplicons were visualized by 1% agarose-gel and compared with a 2-Log DNA ladder (#N3200, New England BioLabs) to confirm size (Figure S2). The PCR amplicons were excised from the gel and purified using the Monarch DNA gel extraction kit (#T1020, New England BioLabs) according to manufacturer’s instructions. Purified amplicons were submitted to GeneWiz (South Plainfield, NJ) for Sanger sequencing. Twenty-three samples (11 from BP, 12 from WL) were sequenced in both directions and the remaining 35 samples were only sequenced in the reverse direction because adequate sequence coverage (872 bp, 77.9% of total cDNA sequence) was achieved using the reverse primer only. Sequence chromatograms were visualized and bases were called manually to generate sequences. Sequences were deposited to NCBI’s GenBank, accession numbers: MT628704-MT6287078. Sequence polymorphisms were identified as double peaks or deviations from the reference sequence (ENSGAFG00000012739). Sequence alignments were performed in A plasmid Editor (ApE, ver. 2.0.55).

**Table 1:** List of primers used for PCR and qPCR in *Gambusia affinis*

Gene	Sequence 5ʹ–3ʹ	Length (bp)
PCR *as3mt* (F)	AGAACCGCTGATAATGGCTCAC	1119
PCR *as3mt* (R)	CGTCCTATGATCATTTGCAGCAG	
qPCR *as3mt* (F)	CGGCTACAAGAAGCCAAACG	148
qPCR *as3mt* (R)	TGCTTCAACCAGAACCTGCT	
qPCR *actb* (F)	GATCTGGCATCACACCTTCTACAA	149
qPCR *actb* (R)	CGTACATGGCAGGAGTGTTGAA	

Accession number for qPCR β-actin (*actb*) primers, AB182330; F forward primer, R reverse primer

### Quantitative PCR

To investigate potential differences in *as3mt* expression among the populations, we conducted quantitative real-time PCR (qPCR) using CFX-Touch 384 real-time thermocycler (BioRad) and SsoAdvanced Universal Inhibitor Tolerant SYBR Green Supermix (#1725017, BioRad). We selected 8, 8, and 16 males each from the BP, SR, and WL (including 10 WL SNP-harboring males) sites. We developed forward and reverse primers (Table 1) over the fourth and fifth exons of reference *G. affinis as3mt* (ENSGAFG00000012739) and also used forward and reverse *actb* (β-actin) primers (Table 1) as a house-keeping reference gene (Huang et al. 2013, 2014). For the experiment, we isolated DNA-free total RNA from individual fish, previously preserved in TRIzol at −80 °C, and synthesized cDNA (as stated above). For real-time quantification, cDNA was diluted 1:5 in nuclease-free H_2_O and 6 μl reactions were performed in triplicate for each cDNA template (each cDNA template represents one fish). Reaction setup followed manufacturer’s instructions, briefly, 1.5 μl cDNA template, 3 μl 2× SYBR Green Supermix, 0.18 μl forward and reverse *as3mt* or *actb* primers [300 nM final], and 1.32 μl DNase-free H_2_O were combined per reaction. Real-time thermocycling was performed according to manufacturer’s suggestions, specifically:

1. Initial denaturation/enzyme activation at 98 °C hold 2 min;
2. 98 °C for 10 sec denaturation, then 60 °C for 20 sec annealing, and extension repeated for 40 cycles, fluorescence emission collected concurrently with extension;
3. Melt curve analysis between 65–95 °C with 0.5 °C increments at 2–5 sec per step.

For each fish, we repeated the real-time PCR on *as3mt* twice with each cDNA template (*i.e*., biological replicate; *n* = 8 BP; *n* = 8 SR; *n* = 16 WL) amplified in triplicate (*i.e*., technical triplicates). The graphed values represent the averages from the two runs (except for four fishes from the BP population, which, due to technical reasons were based on the second run only). Notably, for both repeated runs consistent results within treatment groups were observed, and therefore, we concluded that the second BP assay for these fish was sufficient for reliable analysis. Therefore, the N used for statistical analysis equals the average of the technical replicates (that is, each fish’s average across all technical replicates).

### Statistics

To determine the potential difference in the frequency of any *as3mt* SNP mutations among BP, SR, and WL, we used the Fisher exact test with Bonferroni correction (α = 0.017) as a post-hoc test. To determine the potential difference in *as3mt* expression across genotypes, qPCR data were analyzed using an independent sample t-test because the data were normally distributed (Kolmogorov-Smirnov Normality test, *P* > 0.05). To determine the potential difference in *as3mt* expression across populations, qPCR data were analyzed using a Kruskal-Wallis test because the data were not normally distributed (Kolmogorov-Smirnov Normality test, *P* < 0.05) and the sample size was relatively small. *Post-hoc* multiple comparisons were then followed by a Bonferroni correction. Statistical analyses were done using SPSSPC (ver. 24.0.0.0, IBM SPSS Statistics) and graphs were drawn with Prism (Version 9.00, Prism Graphpad).

## Results

Water parameters are shown in Table 2. Both WL and SR sites had arsenic levels well below the USEPA drinking water limit of 10 ppb (µg/L) as 1.87 ppb and 3.26 ppb, respectively. However, the water from BP site had arsenic levels of 15.72 ppb between 3 and 9 times the levels of the other two sites. The BP site was consistent with previously reported arsenic levels from nearby sites (extracted from data used in Jones et al. 2020); there were no publicly available or published results directly linked to surface water resources in or near the other two sites for comparison. We did not probe for any other potential contaminants in the water samples; and environmental factors such as water temperature were not compared because fishes were collected at different times of year.

**Table 2:** Dissolved arsenic concentration (ppb, μg/L) and pH in the studied three sites (BP Bubbling Pond, SR Salt River, WL Willow Lake)

Population	Arsenic concentration (ppb)			pH
	Average ± SD	RSD	From Jones et al. (2020) Mean ± SD	
BP	15.72 ± 0.22	1.40	16.46 ± 5.9 (*N* = 68)	7.46
SR	3.26 ± 0.30	9.28	N/A	7.73
WL	1.87 ± 0.31	16.66	N/A	7.27

Average and standard deviations (SD) are calculated from technical replicates for each site. Relative standard deviation (RSD) is the absolute value of the coefficient of variation

Eleven out of 24 WL fishes (45.8%), had silent single nucleotide polymorphisms at three different locations in the *as3mt* coding sequence (Table 3). Relative to the reference sequence we observed, nucleotide changes from T to C occurred at nucleotide location 201/1119 and 258/1119 on exon 4 and from G to C at nucleotide location 909/1119 on exon 10 (Figure S3). Three fish did not have the point mutation at the 909 residue; and two fish were homozygotic C at the 201 and 258 residues. The rest of the mutations were all heterozygotic. Unlike fish from the WL population, no fish had any SNPs from BP and SR populations. The occurrence of any *as3mt* SNP mutations was significantly different among the three populations. Specially, the frequency in WL was greater than that in either BP or SR (Fisher exact test, *P* < 0.001).

**Table 3:** Synonymous single nucleotide polymorphism (SNP) in *as3mt* of *Gambusia affinis*, which consists of 1119 bp, having 10 exons and 11 introns: two SNPs on exon 4 and one on exon 10, resulting in four different genotypes of TTG, TTC, CCG, CCC

Ind #	Nucleotide change (location)			Genotype
	T → C (201)	T → C (258)	G → C (909)	TTG
WL3	C>T	C>T	C>G	CCC
WL4	C>T	C>T	C>G	CCC
WL8	C>T	C>T	G	CCG
WL9	C	C	C>G	CCC
WL10	C>T	C>T	G	CCG
WL13	C>T	C>T	C>G	CCC
WL17	T>C	T>C	C>G	TTC
WL18	C	C	C>G	CCC
WL20	C>T	C>T	G	CCG
WL27	T>C	T>C	C>G	TTC
WL31	T>C	T>C	C>G	TTC

**Fig. 3:**
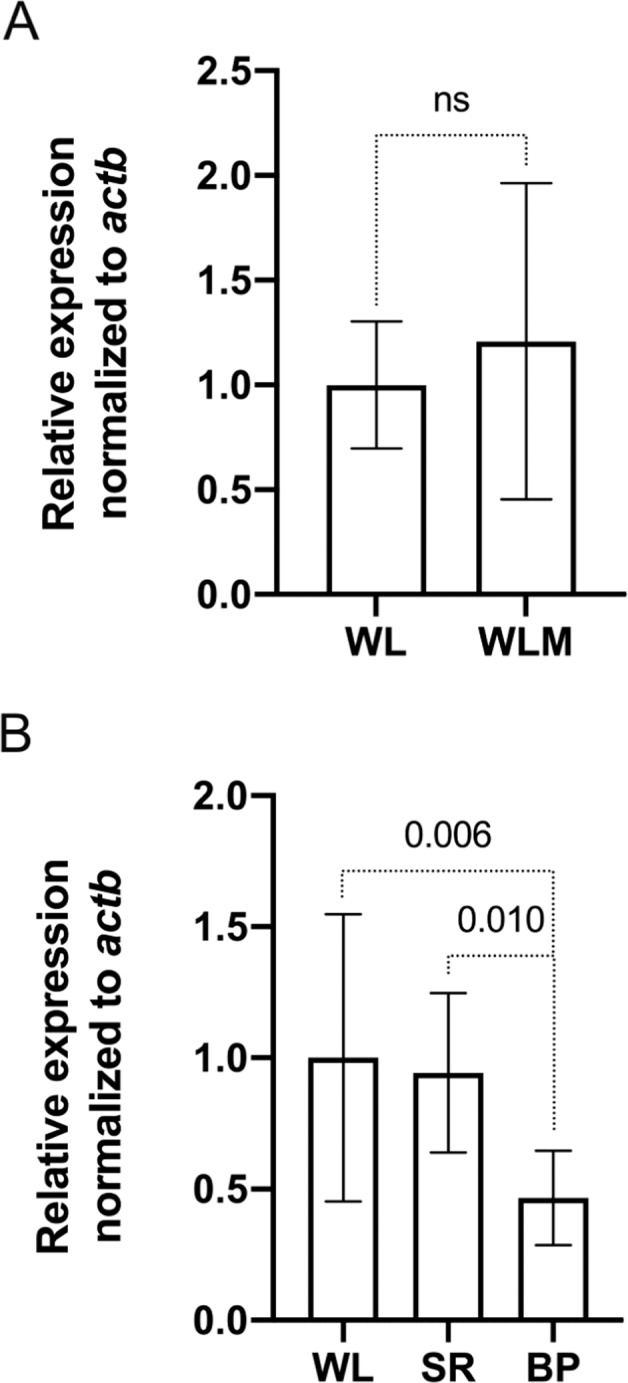
Gene expression of *as3mt* in Western mosquitofishes (*Gambusia affinis*) normalized to *actb* transcript levels. **A** *as3mt* transcript levels in single-nucleotide polymorphic WL fish (WLM) relative to normal WL fish compared by an independent sample *t* test; (**B**) and *as3mt* transcript levels in SR and BP fish relative to WL compared by a Kruskal–Wallis test with *post-hoc* multiple comparisons. Mean and standard deviation are plotted. *P* values are shown above the horizontal lines

The expression of *as3mt* mRNA, normalized to the expression of *actb* mRNA, was not different between WL fish with (WLM) and without (WL) SNPs (*t* = 0.636; *P* = 0.535, Fig. 3A). A significant difference in *as3mt* expression was found among BP, SR, and WL sites (*Kruskal Wallis statistic* = 11.60, df = 2, *P* = 0.003, Fig. 3B). Specifically, the expression of *as3mt* mRNA of BP was approximately 50% lower than that of SR and WL (*P* = 0.01, *P* = 0.006, respectively). While, the expression level between SR and WL mRNA was not different (*P* > 0.05) (Fig. 3B).

## Discussion

### Allelic frequency

To our knowledge, this study provides the first report of any SNP’s in *as3mt* in any vertebrate wildlife species. We found three exonic SNPs on *G. affinis as3mt* in this study. Those SNPs have not been previously reported in humans, and this is the first report of an SNP on exon 4 (Wood et al. 2006; Fujihara et al. 2010; Schlebusch et al. 2013; Antonelli et al. 2014; Agusa et al. 2015; Apata and Pfeifer 2020). Interestingly, the three exonic SNPs in *G. affinis as3mt* found in this study are all synonymous SNPs unlike known human exonic SNPs (Wood et al. 2006). In human populations exposed to high arsenic concentrations over many generations, the frequency of protective mutations is often higher than those seen in populations living with lower levels of exposure, and differences in relative urinary production of MMA and DMA is a representative phenotype of *AS3MT* SNP variation and alterations in enzyme methylation activity (Engström et al. 2007, 2011; Watanabe and Hirano 2013; Antonelli et al. 2014; Apata and Pfeifer 2020). For example, women from populations where water resources contain high arsenic concentrations exhibit population-specific *AS3MT* haplotypes; the haplotypes differ in whether they lead to a higher percentage of MMA or DMA in urine samples (Engström et al. 2011). Other findings in humans, however, show that many other *AS3MT* SNPs identified are neutral, and do not affect detoxification of arsenic (Antonelli et al. 2014). To test the hypotheses that SNPs in the expressed *G. affinis as3mt* gene function to increase arsenic-tolerance, we propose analyzing whole fish arsenic metabolites such as inorganic arsenic, MMA, and DMA from fish with identified differences in SNPs. In addition, verifying more mutations from other non-coding regions of *as3mt* and any potential negative symptoms in morphology and behavior in those SNP *G. affinis* would be worthwhile.

In contrast to our original hypothesis, we found SNPs in *as3mt* among mosquitofish collected from the lowest arsenic contamination level sites. It is possible that the haplotype in the *G. affinis* population from the higher arsenic concentration sites might be adaptive to the high and medium arsenic environments after its initial introduction to the sites, which could result in no SNPs. It is also possible that the *G. affinis* populations in low arsenic water are more tolerant to random mutations because the gene is not under strong selective pressure.

The *as3mt* sequence, also obtained from both BP and SR fishes, is highly conserved across vertebrates including *Danio rerio* and the methyltransferase domain of the *as3mt* has high sequence overlap to parts of functional human *AS3MT* sequences (Palmgren et al. 2017) (Fig. S1). These results imply that *G. affinis as3mt* alleles identified from BP and SR in this study might code for gene products that are functionally efficient in terms of arsenic methylation. Recent studies have found that adaptive mutations occur rapidly in response to chemical (natural and artificial) exposure and lead to local microevolutionary outcomes (Hamilton et al. 2017; Gouin et al. 2019) support this interpretation. Because two of our study populations had gene sequences similar to each other and to the zebrafish genome, our results suggest that SNPs identified in the Willow Lake population occurred over the relatively short period since their introduction to this region (Dees 1961; Miller and Lowe 1967) and future studies can investigate whether these differences in *as3mt* haplotypes provide protection against the toxic effects of arsenic exposure.

### *as3mt* expression

*Gambusia affinis* expressed *as3mt* at the same levels independently of genotype, suggesting that these SNPs do not decrease or increase *as3mt* expression. Furthermore, the synonymous nature of the SNPs would be unlikely to change protein properties. This expression level of *as3mt* may be sufficient to provide a protective phenotype, especially in a low arsenic environment such as was found in this population. In humans, increased *as3mt* expression is more evident when SNPs were deleterious, presumptively as a compensatory mechanism (Engström et al. 2011). Lastly, SNPs might affect overall *as3mt* functions through post-transcriptional processes rather than through gene transcription. In human *MDR1* (multidrug resistance 1), silent SNPs post-transcriptionally affected timing of co-translational folding, resulted in altered structure of substrate and inhibitor interaction sites (Kimchi-Sarfaty et al. 2007). In humans, nonsynonymous *as3mt* SNPs also changed arsenic-affinity and stability of *as3mt* through conformational structure changes of proteins, but not by changing gene expression (Li et al. 2017).

Mosquitofish *as3mt* expression was significantly lower in the site containing the highest arsenic concentrations, which was opposite to our initial expectation. This result suggests *as3mt* expression in *G. affinis* at this site might be under control of upstream regulatory processes. For example, in humans, DNA-methyltransferase is directly linked with *as3mt* expression (Engström et al. 2011). Also, we have not evaluated the promotor region of the gene for fish from these populations. Nevertheless, this lower *as3mt* expression in BP population compared to the WL and SR populations living in waters with lower arsenic concentrations suggests fish in BP population may exhibit signs of toxicity. Future studies will test the ratio of whole-body MMA/DMA in fish from these populations, investigate whether there are SNPs in the promotor region of a*s3mt*, and evaluate these populations for other arsenic-related morphological characteristics such as curved spines (Gonzalez et al. 2010; Sun et al. 2016).

## Conclusion

In summary, this preliminary study provides the first evidence of sequence variation in *as3mt* in a wildlife population and has several interesting implications for the evolution of toxic responses in wildlife. We found significantly different SNP frequency in *as3mt* across different arsenic-exposure populations of Western mosquitofish, but *as3mt* expression was not different between normal and SNP carrying fishes. The results were not consistent with our original hypothesis that *G. affinis* exposed to high arsenics could show greater SNP frequency and higher *as3mt* expression. Future studies will determine whether there are morphological arsenic-related symptoms across populations. In addition, future studies could evaluate mutations in both the promotor and intron regions of *as3mt*. Because of their wide global distribution, Western mosquitofish may prove to be a good model to study the complexity of microevolutionary processes in response to arsenic exposure.

## Supplementary information

Supplementary Figures

Supplementary Information

## Data Availability

Sequences obtained herein have been deposited to NCBI. Collecting sites for mosquitofish have been identified.
