# Supplementary material for: Synonymous single nucleotide polymorphism in arsenic (+3) methyltransferase of the Western mosquitofish (Gambusia affinis) and its gene expression among field populations
Source: Ecotoxicology. 2021 Apr 3;30(4):711–8. doi: 10.1007/s10646-021-02376-8 (PMC8060185; doi:10.1007/s10646-021-02376-8)

**Supplementary figures**

**Figure S1.** Primary sequence alignment of AS3MT proteins from human, mouse, chicken, *Xenopus*, zebrafish, and mosquitofish using Clustal Omega (Sievers et al. 2011).


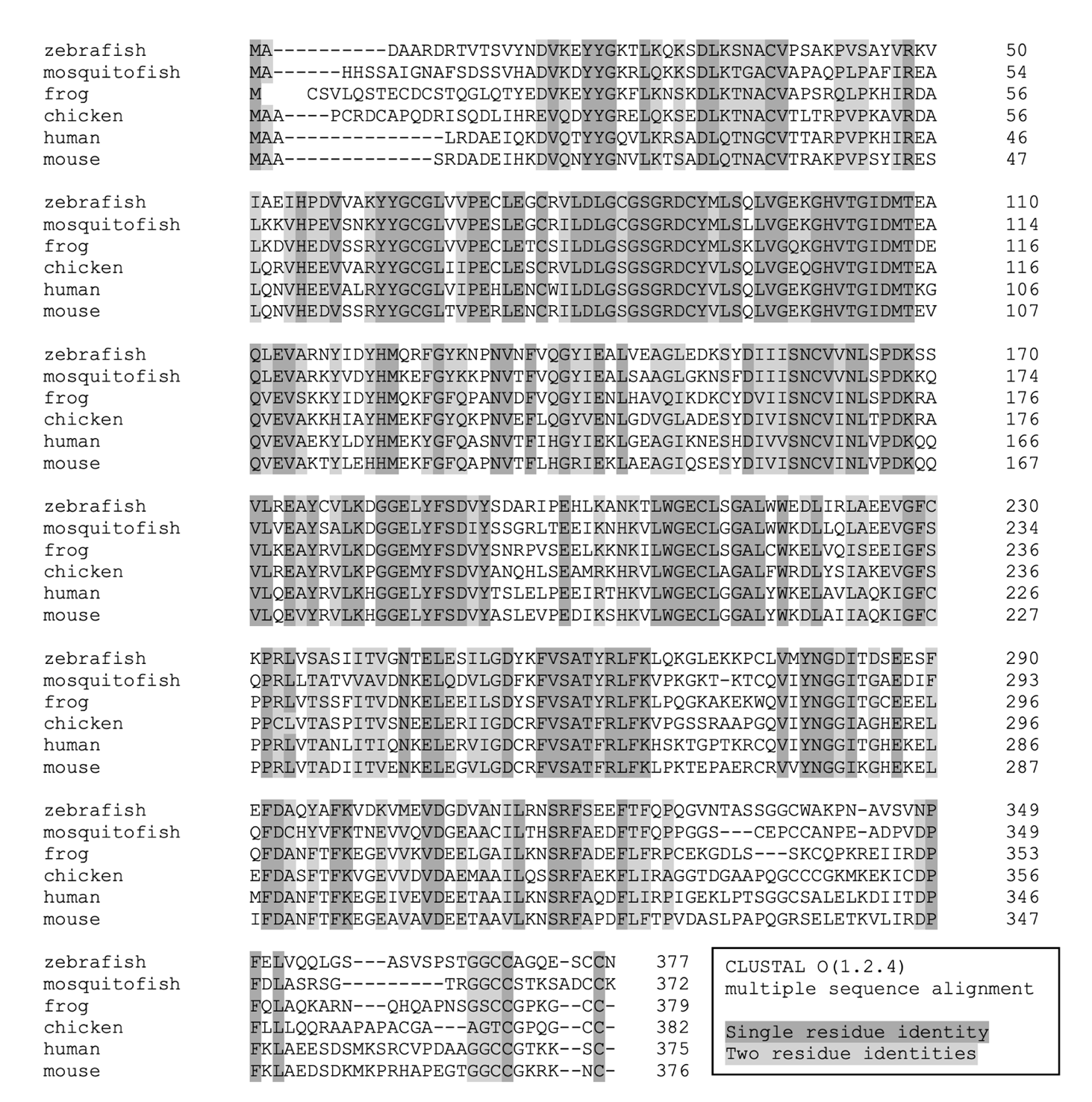


**Supplementary Reference**

Sievers F, Wilm A, Dineen D et al. (2011) Fast, scalable generation of high-quality protein multiple sequence alignments using Clustal Omega. Mol Syst Biol 7:539. <https://doi.org/10.1038/msb.2011.75>

**Figure S2.** PCR amplicons (1119 bp) on 1% agarose gel: from WL#17 to WL#24.


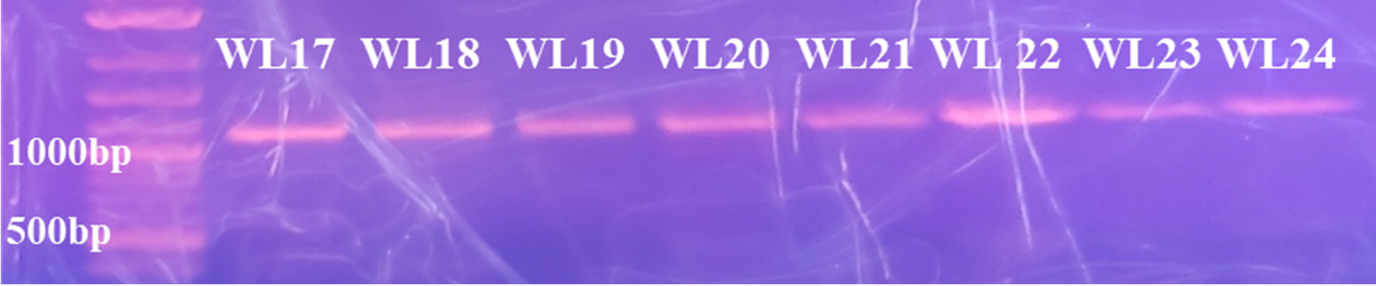


**Figure S3.** Chromatographs, showing different SNPs at 201, 258, and 909 sites, where T🡪C, T🡪C, and G🡪C mutations occur, respectively, resulting in four different genotypes. Forward primer was used for 201 and 258 site, while reverse primer was used for 909 site. BP#6 (normal fish): TTG type, WL#31: TTC type, WL#20: CCG type, WL#18: CCC type (See Table 3).


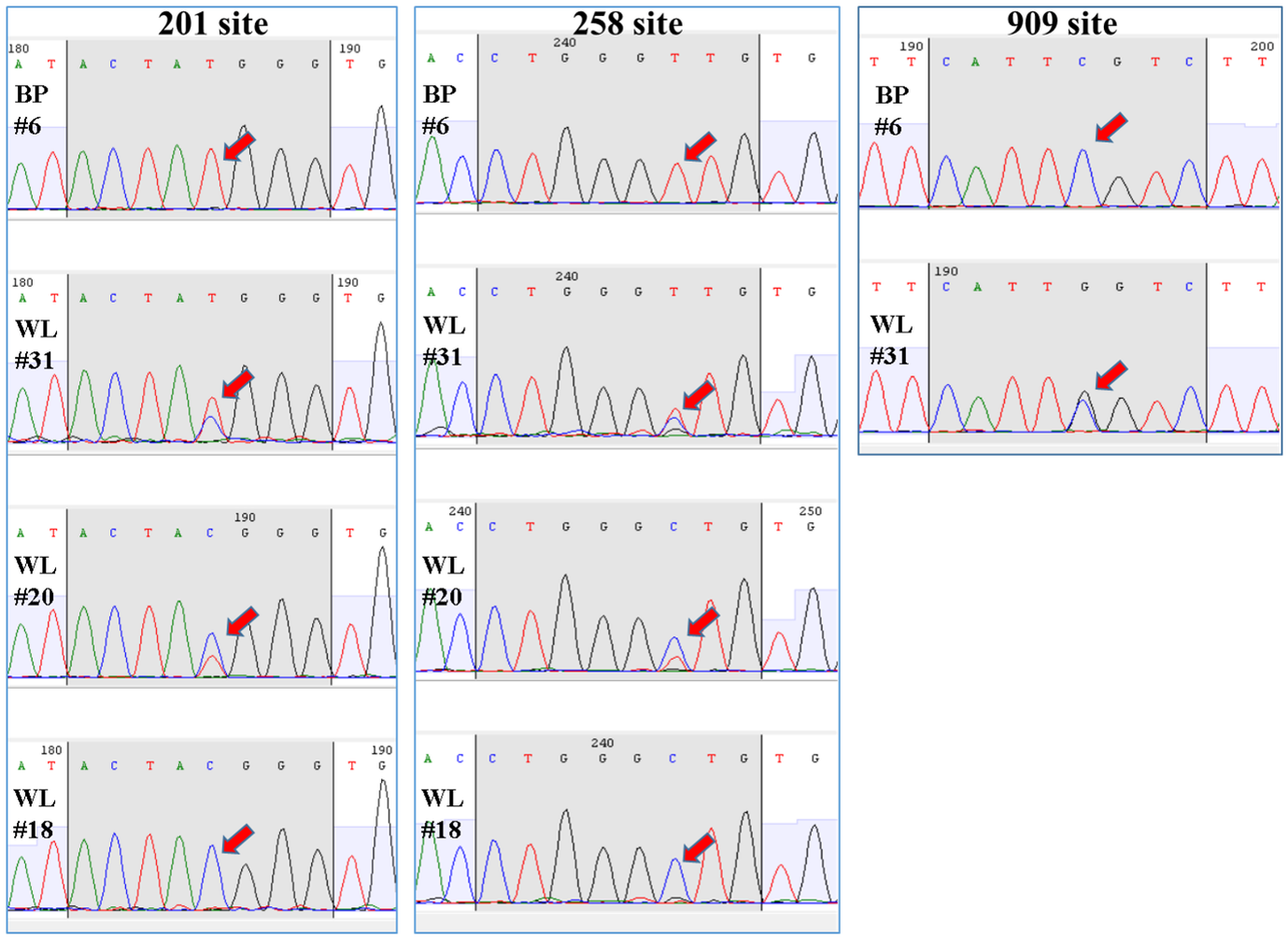

Supplement: Supplementary file 1 — Supplementary Figures [file 10646_2021_2376_MOESM1_ESM.docx]
